# Occurrence of ESBL-Producing *Escherichia coli* in Livestock and Farm Workers in Mecklenburg-Western Pomerania, Germany

**DOI:** 10.1371/journal.pone.0143326

**Published:** 2015-11-25

**Authors:** Carmen Dahms, Nils-Olaf Hübner, Annelene Kossow, Alexander Mellmann, Kathleen Dittmann, Axel Kramer

**Affiliations:** 1 Institute of Hygiene and Environmental Medicine, University Medicine Greifswald, Greifswald, Germany; 2 Institute of Medical Diagnostics (IMD), Greifswald, Germany; 3 Institute of Hygiene, University Hospital Münster, Münster, Germany; Justus-Liebeig University Giessen, GERMANY

## Abstract

In recent years, extended-spectrum β-lactamases (ESBL) producing bacteria have been found in livestock, mainly as asymptomatic colonizers. The zoonotic risk for people working in close contact to animal husbandry has still not been completely assessed. Therefore, we investigated the prevalence of ESBL-producing *Escherichia* spp. in livestock animals and workers to determine the potential risk for an animal-human cross-transmission.In Mecklenburg-Western Pomerania, northeast Germany, inguinal swabs of 73 individuals with livestock contact from 23 different farms were tested for ESBL-producing *Escherichia* spp. Two pooled fecal samples per farm of animal origin from 34 different farms (17 pig farms, 11 cattle farms, 6 poultry farms) as well as cloacal swabs of 10 randomly selected broilers or turkeys were taken at each poultry farm. For identification, selective chromogenic agar was used after an enrichment step. Phenotypically ESBL-producing isolates (n = 99) were tested for CTX-M, OXA, SHV and TEM using PCR, and isolates were further characterized using multilocus sequence typing (MLST). In total, 61 diverse isolates from different sources and/or different MLST/PCR results were acquired. Five farm workers (three from cattle farms and two from pig farms) harbored ESBL-producing *E*. *coli*. All human isolates harbored the CTX-M β-lactamase; TEM and OXA β-lactamases were additionally detected in two, resp. one, isolates. ESBL-producing *Escherichia* spp. were found in fecal samples at pig (15/17), cattle (6/11) and poultry farms (3/6). In total, 70.6% (24/36) of the tested farms were ESBL positive. Furthermore, 9 out of 60 cloacal swabs turned out to be ESBL positive. All isolated ESBL-producing bacteria from animal sources were *E*. *coli*, except for one *E*. *hermanii* isolate. CTX-M was the most prevalent β-lactamase at cattle and pig farms, while SHV predominated in poultry. One human isolate shared an identical MLST sequence type (ST) 3891 and CTX-M allele to the isolate found in the cattle fecal sample from the same farm, indicating a zoonotic transfer. Two other pairs of human-pig and human-cattle *E*. *coli* isolates encoded the same ESBL genes but did not share the same MLST ST, which may indicate horizontal resistance gene transfer. In summary, the study shows the high prevalence of ESBL-producing *E*.*coli* in livestock in Mecklenburg- Western Pomerania and provides the risk of transfer between livestock and farm workers.

## Introduction

Extended- spectrum β-lactamases (ESBL) are mainly plasmid-encoded enzymes providing an extended resistance to β-lactam antibiotics and can be produced by a variety of different bacteria, such as Enterobacteriaceae or nonfermenting bacteria [1, 2]. *Escherichia coli* (*E*.*coli*) and *Klebsiella pneumoniae* are the most frequently found ESBL-producing bacteria [2] and may cause urinary tract infections (UTI), pneumonia or even sepsis [2, 3]. CTX-M β-lactamases have emerged as the most prevalent ESBL enzymes in humans [4], whereas subtypes varying depending on the geographic region [5]. Since over three decades ESBL-producing bacteria were known as nosocomial pathogens and since the late 1990s they were increasingly found as a causal agent of infections in the community [6].

The occurrence of ESBL-producing bacteria has been broadly recognized in veterinary medicine, e.g. as causative agents for mastitis in dairy cattle [7] since the 2000s [8, 9], but only a few studies exist which investigated the prevalence of ESBL-producing bacteria in German livestock, showing their existence in sick and healthy cattle, pig and poultry farms [10–14]. The CTX-M group, in Europe especially CTX-M-1 β-lactamase, is the most frequently detected ESBL in livestock [5]. The risk of zoonotic transfer from livestock to people with close contact to these animals is still largely unknown, but some studies have implicated a transfer of ESBL-producing *E*. *coli* or ESBL genes from poultry or pigs to farm workers [15–18]. Besides this direct zoonotic transfer, other routes as foods of animal origin may be a risk factor for human colonization or infection [19, 20]. However, ESBL-producing bacteria are not only found in livestock, but also in companion animals [8, 21], zoo animals [22] and wild animals [23–26].

In our study, we assessed the occurrence and zoonotic potential of ESBL-producing bacteria by comparison of the ESBL genes and sequence types (STs) of multilocus sequence typing (MLST) in farm workers as well as at pig, cattle and poultry farms in the region of Mecklenburg-Western Pomerania (MP) in northeastern Germany.

## Material and Methods

### Design

We conducted a cross-sectional study of the occurrence of ESBL-producing bacteria in people who were in close contact with farm animals in comparison to the ESBL prevalence in livestock in MP. The design of this study was approved by the ethics committee of the Ernst Moritz Arndt University of Greifswald in February 2012 (No.BB 07/12).

From March to June 2012, 73 people (31 female, 42 male) with livestock contact from 23 farms in MP were screened for ESBL-producing bacteria. 32 participants worked at pig farms, 24 at cattle farms and 17 persons worked at poultry farms (Table 1). Two of the 73 participants were included in the study despite not being employed farm workers, as they were in regular close contact with farm animals.

**Table 1:** Numbers and results of participant farm workers and farms.

farm	participant farm workers	*(%)*	ESBL- positive workers	*(%)*	participant farms	*(%)*	ESBL_-_positive farms	*(%)*
cattle	24	*(32*.*9)*	3	*(12*.*5)*	11	*(32*.*4)*	6	*(54*.*5)*
pig	32	*(43*.*8)*	2	*(6*.*3)*	17	*(50*.*0)*	15	*(88*.*2)*
poultry								
broiler	8	*(10*.*9)*	0	*(0)*	4	*(11*.*7)*	3	*(75)*
turkey	9	*(12*.*3)*	0	*(0)*	2	*(5*.*9)*	0	*(0)*
**total**	**73**	***(100)***	**5**	***(6*.*8)***	**34**	***(100)***	**24**	***(70*.*6)***

A total of 34 farms were included: 17 pig, 11 cattle and 6 poultry farms (4 broiler, 2 turkey farms) and fulfilled the following criteria: the number of animals should be at least 50 pigs or cattle, or 10.000 poultry (only fattening) and the localization preferentially in the northeastern part of MP. Four of the pig farms were organic. On poultry farms, cloacal swabs were additionally taken from 10 randomly chosen animals per farm. All participants gave written informed consent after clarification of the purposes of the study.

### Sampling

The participants themselves took inguinal samples with a sterile swab (Amies transport medium, transystem®, Copan Italia *Spa*, Brescia, Italy) according to instruction by trained physicians or veterinarians.

Two pooled fecal samples per cattle or pig farm were separately screened for ESBL-producing bacteria. Each sample weighed at least 25 g and represented the feces of at least ten animals. At poultry farms, boot swabs from one to two barns were collected by walking at least half the length of the tested barn. Additionally, 10 randomly selected animals were sampled by taking cloacal swabs (Amies transport medium, transystem®, Copan Italia *Spa*, Brescia, Italy) by a veterinarian. The animals were neither hurted nor injured by this procedure. Samples were processed within 24 hours to 48 hours.

### ESBL isolation

The processing of human isolates was performed in laboratory of the Institute of Hygiene and Environmental Medicine in Greifswald, the feces samples and boot swabs were processed at the Federal state laboratory of MP and the poultry swabs were processed at the Robert Koch Institute, Berlin.

Human inguinal swabs were directly streaked onto Brilliance^TM^ ESBL Agar (Oxoid, Basingstoke, UK). Additionally, the swab was squeezed in Tryptic Soy Broth (Bacto™ Tryptic Soy Broth, Becton & Dickinson, Le Pont de Claix, France) and after incubation at 37±1°C for 18–24 hours, 10 μl were streaked on Brilliance^TM^ ESBL Agar. After 24 and 48 hours incubation, the presumptive colonies were identified by growth on the chromogenic screening plate. For confirmation of the ESBL production, disk diffusion tests were performed using disks loaded with either Cefotaxim 5 μg or Ceftazidim 10 μg (both Oxoid, Basingstoke, UK) according to EUCAST breakpoints (http://www.eucast.org/clinical_breakpoints/) as well as E-Test with Cefotaxim/Clavulanic acid and Ceftazidim/Clavulanic acid (both bioMérieux, Marcy l’Etoile, France). The inhibition by clavulanic acid, a ß-lactamase inhibitor, indicated the presence of an ESBL enzyme.

For detection of ESBL in animal samples, the feces, boot and cloacal swabs, were mixed into buffered peptone water (Peptone, disodium hydrogene phosphate and monopotassium phosphate from Merck KGaA, Darmstadt, Germany; sodium chloride from SIFIN, Berlin, Germany) at a 1:9 ratio and incubated at 35–37°C for 18–24 hours. Afterwards, 10 μl were streaked onto Brilliance^TM^ ESBL Agar (Oxoid, Basingstoke, UK) and incubated at 35–37°C for 18–24 hours.

For confirmation of ESBL production, here the Cefpodoxime Combination Disc Kit (Oxoid, Basingstoke, UK) was used according to manufacturer’s instructions. From fecal samples and inguinal swabs each morphologically different colony was selected. In contrast, five colonies were randomly chosen for further investigation from cloacal samples. All isolates were stored at -20°C in cryobank tubes according to the instructions of the manufacturer (Mast CRYOBANK™, Reinfeld, Germany).

### Genotypic characterization

Of all ESBL-producing *Escherichia* spp. isolates, the species was confirmed using matrix-assisted laser desorption/ionization-time-of-flight mass spectrometry (MALDI-TOF MS) before they were further characterized using a PCR assay for the detection of ESBL encoding genes. To elucidate their phylogenetic background MLST was performed using the *E*. *coli* MLST scheme published by Wirth et al. [27]. For both assays, DNA was extracted as follows: a single bacterial colony was transferred into 100 μl 5% Chelex® 100 Resin (Bio-Rad Laboratories, Hercules, CA, USA), boiled for 10 min at 100°C and centrifuged for 2 min at 12,000 g. Subsequently, 60 μl of the supernatant including the DNA were transferred into a new reaction tube and used as PCR template in a 1:100 dilution. For detection of the β-lactamase genes (*bla*_CTX-M_, *bla*_OXA_, *bla*_SHV_, *bla*_TEM_), a multiplex PCR was used as published [28]. Additionally, for the human isolates and the corresponding livestock isolates Sanger sequencing of the PCR products was performed to enable further subtyping of the ESBL genes. Resulting partial sequences of the ESBL genes were blasted against the published ESBL reference sequences and alleles were named in accordance to http://www.lahey.org/studies/. In case of ambiguous results, all possible ESBL alleles were given; if sequencing failed, the alleles were rated as “non-typeable”.

For MLST, the previously described protocol by Wirth et al. was applied [27]. Resulting sequences of the seven house-keeping genes were analyzed using the SeqSphere^+^ software version 1.0 (Ridom GmbH, Münster, Germany) and MLST sequence types (ST) and clonal complexes (CC) were assigned in accordance to the *E*. *coli* MLST website (http://mlst.warwick.ac.uk/mlst/dbs/Ecoli). Phylogenetic trees were constructed using SeqSphere^+^ software (Ridom GmbH).

### Data collection

Information about age, sex and the average daily working hours (based on a 5-day working week) of the farm workers as well as the specialization of the farm (e.g., breeding farm, dairy farm) and whether it was organic or conventional were recorded. All collected data were electronically stored and evaluated using IBM SPSS Statistics 22 to compare farm workers working in farms with an ESBL-positive fecal sample carrying ESBL-producing bacteria with non-carriers for significant differences in these parameters. Mann-Whitney-U-Test and Fisher’s exact test were used and p-value cut-off was set as ≤ 0.05.

## Results

### Microbiological analysis

In total, 99 isolates were obtained and further analyzed. Of those, 38 isolates were of supposed clonal origin, as they were found in the same sample source and showed identical MLST and PCR results. Therefore, 61 distinct isolates from human (n = 5), cattle (n = 10), pig (n = 33) and poultry (n = 13) sources were included in the assessment.

Five of 73 (6.8%) farm workers carried ESBL-producing *E*. *coli* and all were employed at farms with positive livestock fecal samples (Table 1). Two individuals worked on pig farms and three on cattle farms. One of the two pig farms was a mixed breeding/ rearing farms, the other farm was a breeding farms. Two of three cattle farm workers carrying ESBL-positive *E*. *coli* worked at the same farm. This farm was a mixed dairy cattle and fattening farm, the other cattle farm kept only dairy cattle. No significant differences in sex, age or average daily working hours between the ESBL-positive and ESBL-negative farm workers could be detected (Table 2).

**Table 2:** Comparison farm workers working in farms with an ESBL-positive fecal sample (n = 55)* carrying ESBL-producing bacteria with non-carriers.

	ESBL-carrier (n = 5)	ESBL non-carrier (n = 50)	Significance
**Mean age in years [95% CI]**	4.4 [38.7–56.1]	46.5 [42.7–50.2]	p = 0. 943
**Sex**	3 female; 2 male	23 female; 27 male	p = 0.447
**Mean daily work in hours [95% CI]**	9.2 [7.2–11.2]	6.5 [5.4–7.5]	p = 0.127

* only people working in cattle farms with an ESBL-positive result were included. No significant differences could be detected.

In 15 of 17 (88.2%) participating pig farms, ESBL-producing bacteria in fecal samples were detected (Table 1). Four of them were organic farms. The majority of farms (n = 9) were mixed farms with breeding, rearing and fattening. Altogether, 33 ESBL-producing isolates were found in pig farms. Six of the 33 isolates were from organic farms. All isolates were identified as *E*. *coli*, except for one *E*. *hermanii* isolate. Due to its close relation to *E*. *coli*, the *E*. *hermanii* isolate was included in the study.

Six of 11 cattle farms (54.5%) were positive for ESBL-producing *E*. *coli* (Table 1). All cattle farms kept dairy cattle; three farms also had suckling calves and/or beef cattle. In total, ten ESBL-producing isolates were found.

Three of six poultry (50%) farms turned out to be positive for ESBL-producing *E*. *coli*, all of which were broiler farms (Table 1). Three isolates were found. Nine of the 60 cloacal swabs harbored ESBL-producers and ten isolates were obtained. In two of three broiler farms with ESBL-positive boot swabs, ESBL-producing isolates from animals were detected via cloacal swabs. From one farm with an ESBL-positive boot swab sample, only one of ten cloacal samples was ESBL-positive. From another ESBL-positive broiler farm, eight cloacal samples were positive for ESBL-producing *E*. *coli*. In one broiler sample two isolates were found.

### Phylogenetic analysis

**Fig 1:**
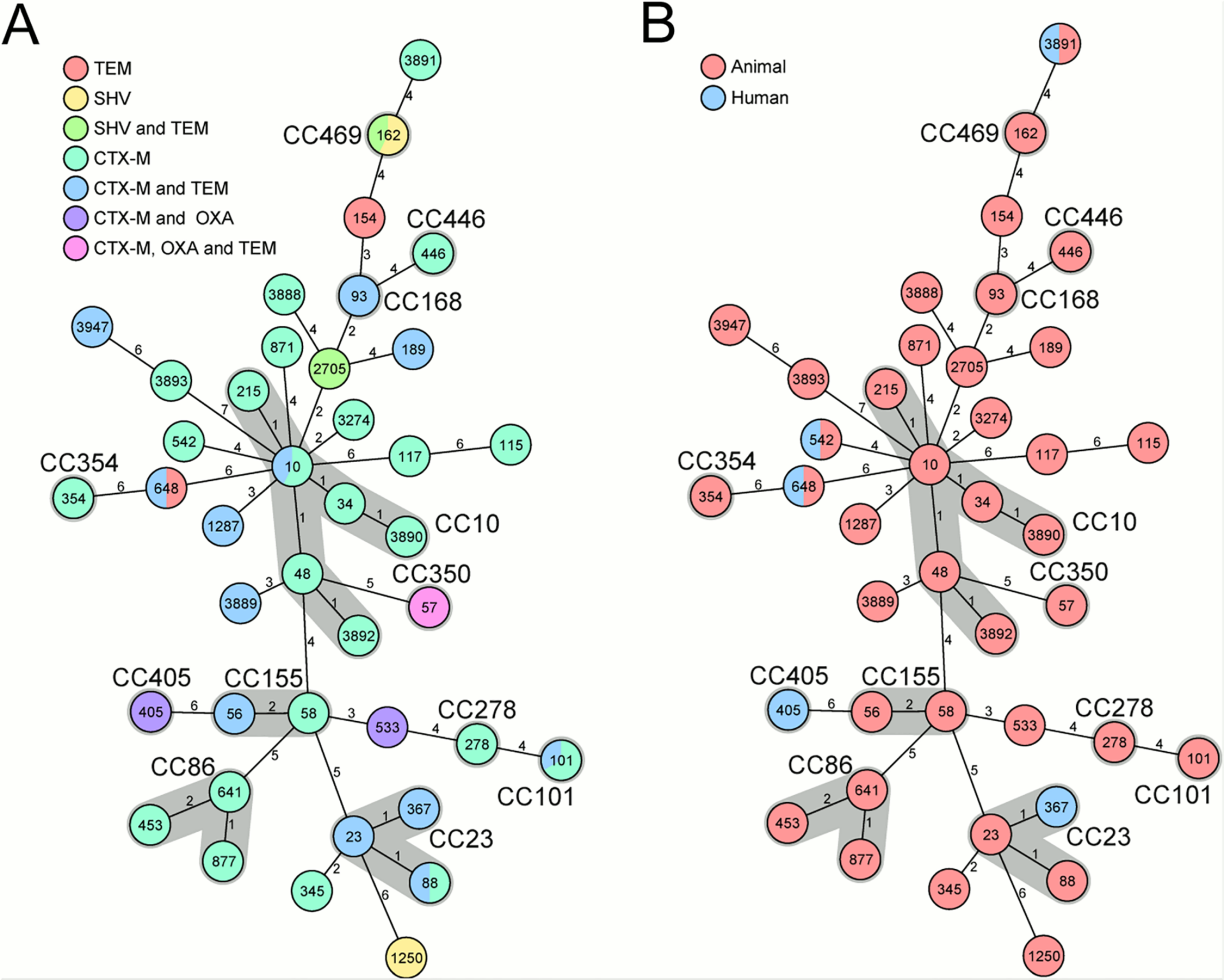
Minimum spanning trees based on MLST allelic profiles portraying the clonal relationship of ESBL-producing E. coli. Each circle represents a given allelic profile (combination of the seven MLST loci) and is named according to the MLST sequence type. The numbers on the connecting lines illustrate the number of differing alleles. If applicable, the clonal complexes (CC) are shaded in grey and named. The correlations between the MLST-based phylogeny and (1A) ESBL genotype represented by the differently colored circles and (1B) origin (animal or human) of the samples are displayed.

Genotypic analysis of the investigated strains (n = 61) showed a diverse collection of ESBL-producing isolates. Overall, 40 different STs and 13 different CCs were detected; among these, ST162 (CC469, n = 11) were the most common (Fig 1). Fig 1A shows the correlation between the MLST-based phylogeny and the distribution of ESBL genes. Whereas the majority of STs only carried the same ESBL gene(s) or combination of genes, five STs, namely STs10, 88, 101, 162, 648, had two different combinations of ESBL encoding genes.

**Fig 2:**
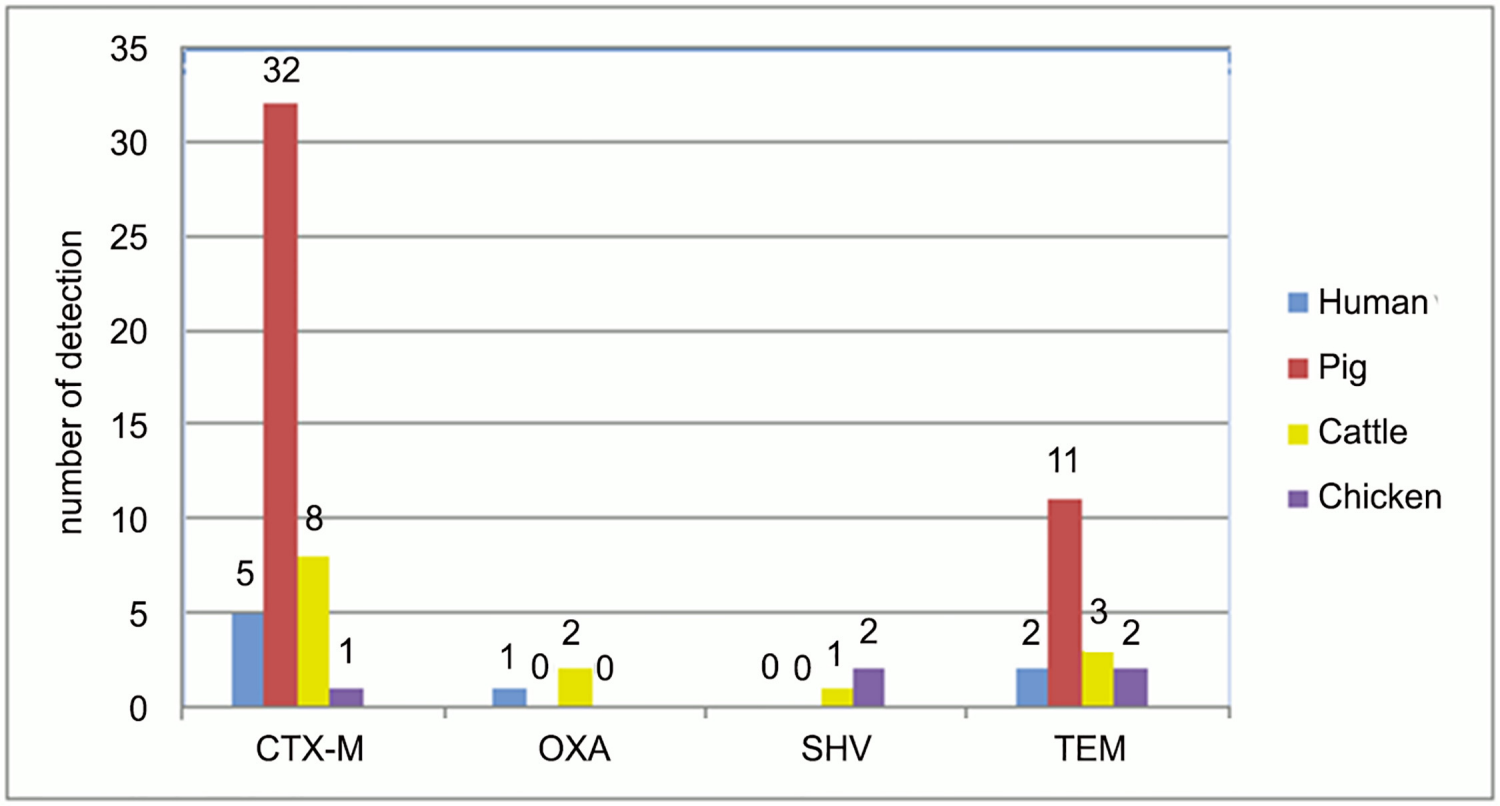
Distribution of ESBL genes in samples of human, pig, cattle and chicken origin. The figure shows the distribution of the ESBL genes CTX-M, OXA, SHV and TEM of *Escherichia* spp. isolates from humans (inguinal swabs), pig and cattle (fecal samples) and broiler (boot swabs). CTX-M ESBL dominated in pigs with 32 isolates; 8 were found in cattle, 5 in humans and 1 in poultry. OXA enzymes were rare, but most frequently isolated from cattle (n = 2). SHV were also rare and dominated in poultry (n = 2). TEM were most frequently found in isolates of pig origin (n = 11).

All human isolates (n = 5) were positive for CTX-M, one human sample was positive for OXA, too. The isolates of the two pig-farm workers were also positive for the TEM group. Fig 1B illustrates the *E*.*coli* MLST CC and ST, Fig 2 the ESBL genes found in humans and livestock.

At pig farms, 27 different STs were found. The β-lactamase CTX-M (n = 26) was always detectable, except for one isolate which was only positive for the TEM β-lactamase. Ten isolates determined for CTX-M and TEM enzymes.

At cattle farms, 8 of 10 isolates found produced CTX-M β-lactamases, two of them carried also the *bla*_OXA_ gene and one of them harbored a third β-lactamase, the TEM enzyme. One isolate had both CTX-M and TEM β-lactamases. Another isolate carried only the SHV β-lactamase and another had only the TEM enzyme. Nine different STs were detected; ST446 was identified in isolates from two different farms.

At the poultry farms, one boot swab isolate contained a CTX-M β-lactamase (ST115). The other two isolates contained SHV and TEM β-lactamases.

In one broiler farm, the cloacal and the corresponding boot swab sample carried the same ESBL gene (SHV), but represented different ST types (ST162 and ST2705). In the second farm with an ESBL-positive boot sample and corresponding cloacal swabs, all isolates (n = 10) also belonged to ST162 (CC469). All were positive for SHV and five were additionally positive for TEM β-lactamase. An ESBL variant with the *bla*_SHV_ and *bla*_TEM_ genes and a variant with only the *bla*_SHV_ gene were detected in the same animal.

### Comparison human and livestock results

In Fig 1B, the distribution of human and animal sample origin is displayed. In three STs (ST542, ST648, ST3891), the MLST genotype is shared by human and animal isolates; however, only two CTX-M-positive ST542 and ST3891 were present in humans and animals (Fig 1). Of these, one human isolate harbored the same ST3891 variant and subtyping of the CTX-M β-lactamase using partial sequencing of *bla*_CTX-M_ resulted in two possible alleles, CTX-M-1 and CTX-M-61 ESBL (S1 Table), which was also found in the corresponding fecal sample from the same farm. One pair of isolates (human and cattle) also shared CTX-M-1/-61, but exhibited different STs (ST542 and ST533). The cattle isolate encoded also an OXA ß-lactamase, where partial sequencing of the PCR product failed, likely due to different copies of the gene or due to frame shift mutations (“non-typeable”). In this farm, a second farm worker harbored an ESBL-producing *E*. *coli*, encoding a CTX-M-15/-28/-88 and OXA β-lactamase which was also non-typeable. One pair (human and pig) of isolates also shared CTX-M-1/-61 and TEM-104/-206 ESBLs with different STs (ST367 and ST10). The isolate of another pig farm worker encoded a TEM-104/-206 ESBL and the CTX-M encoding genes were non-typeable. Four different ESBL-producing isolates were found in the corresponding pig feces samples. Two isolates encoded the CTX-M-1/-61 ESBL, in one isolate the CTX-M encoding genes were non-typeable and the fourth isolate encoded the CTX-M1/-61 as well as the TEM-104/-206 ESBL. The STs of these human and pig isolates differed as shown in Table 3.

**Table 3:** Overview of the ESBL-PCR and MLST-results of the human-livestock isolates.

		CTX-M-1/-61	CTX-M-15/-28/-88	CTX-M (non-typeable)	TEM-104/-206	OXA (non-typeable)	
Farm	Isolate	ESBL genes *					MLST ST
**No. 1**	worker 1	X					ST3891
	cattle 1	X					ST3891
**No. 2**	worker 2a		X			X	ST405
	worker 2b	X					ST542
	cattle 2	X				X	ST533
**No. 3**	worker 3	X			X		ST367
	pig 3	X			X		ST10
**No. 4**	worker 4			X	X		ST648
	pig 4a	X			X		ST3947
	pig 4b			X			ST3893
	pig 1c	X					ST88
	pig 1d	X					ST453

* due to partial *bla* sequencing, closely-related alleles could not be discriminated as the covered region was identical in all alleles. Therefore, all possible alleles were given here. In case of sequencing failure, the alleles were rated as “non-typeable”.

## Discussion

The direct transfer from ESBL-producing bacteria to humans via close contact seems possible, but evidence is sparse [5, 29]. Wu et al. (2013) used MLST and virulence and resistance gene microarrays to investigate ESBL-producing *E*. *coli* from human and animal sources from Germany, the Netherlands and the UK. Merely 1.2% of the animal isolates shared the same MLST CC with the human ones indicating that zoonotic transmission plays no or a minor role [30]. A review by Ewers et al. (2012) reached a similar conclusion, as the main ESBL subtypes vary distinctly in human and livestock sources [5]. In contrast, ESBL-producing *E*. *coli* isolates from German livestock and humans recently showed a higher occurrence of CTX-M-1 in humans (29.3%) and CTX-M-15 ESBL in livestock (17.7%) than did the review by Ewers et al. (7% and 8%, resp., for Europe) [5, 31]. Furthermore, similar ESBL isolates were found in human isolates and in poultry meat samples in the Netherlands, also suggesting a zoonotic transfer [19, 32]. A direct transfer from poultry to people with close contact seems possible as well [17]. Huijbers et al. revealed an increased risk for people with close broiler contact as well as strong indications of the transmission from animals [18]. Pigs may also play a significant role as vectors: *E*. *coli* isolates from Danish pigs and pig farm workers harbored the same CTX-M-1 carrying plasmids, indicating a horizontal transfer of plasmids between *E*. *coli* lineages from livestock and human sources [15]. Recently, another Danish study strengthened these results, finding highly similar ESBL-producing *E*. *coli* in pigs and pig farm workers [16].

In our study, all farm workers carrying ESBL-producing *E*.*coli* worked on positive-tested farms, and two positive-tested cattle farm workers were employed at the same farm. Furthermore, three farm workers with ESBL-producing *E*.*coli* harbored the same MLST ST that was also found in livestock sources. The isolates from one human and one cattle source had the identical MLST result and revealed a CTX-M-1/-61 β-lactamase. This suggests that the isolates descend from the same *E*. *coli* clone. Unfortunately, our assay was not able to distinguish between CTX-M-1 and -61 allele because of their great similarity as they differ only by one amino acid. CTX-M-61 is rarely found in Enterobacteriaceae [33, 34], whereas the CTX-M-1 β-lactamase is the most frequently isolated CTX-M allele from livestock sources in Europe, but can also be occasionally found in humans [5]. Furthermore, the same ESBL genes were found in human and livestock isolates, which may indicate a horizontal resistance gene transfer, as ESBL are often plasmid-encoded. Characterization of the plasmids or whole genome sequencing would yield more precise results.

Finding the same ESBL-producing *E*.*coli* in livestock and farm workers does not prove a zoonotic transfer. A common source of transmission is also conceivable as epidemiological link. Generally, livestock may spread ESBL-producing bacteria into the environment, e.g., via ESBL-positive slurry used as fertilizer for crop farming [10]. In consequence, resistant bacteria may disseminate into the soil [35] and the ground water and dissemination through the air, rodents or other vermin is conceivable [13, 36–38]. Antibiotics and resistant bacteria can also be distributed through human sources, e.g., through waste water [39].

Due to small sample sizes, regional concentration and voluntary participation of the tested farms and farm workers, our study is not meant to be representative for the prevalence of ESBL-producing bacteria in MP. The number of positive farm workers found in our study was rather small. The prevalence of ESBL-producing *E*. *coli* in the general community is still not completely assessed. In the Netherlands, 4.9% (45/927) of the community harbored ESBL-producing *E*. *coli* at admission screening [32] and recently, a study revealed a prevalence of 6.3% (211/3344) in Bavaria, Germany [40]. Compared to our results, the difference is quite small, but the number of participants in our study is rather low and we did not analyze rectal swabs or fecal samples. This would have been more sensitive; however, rectal swabs are typically associated with low compliance. Furthermore, the pre-enrichment was not selective for ESBL-producers, adding a cephalosporine could have improved the detection rate [41]. Therefore, an underestimation of the ESBL-producing bacteria is very likely. Likewise, investigating only one morphologically different colony growing on the chromogenic agar plate could also led to a underestimation of existing ESBL genes and MLST STs, otherwise, the five isolates from the poultry cloacal swabs were almost all clones of the same *E*. *coli*. It was already shown that the number of isolates tested within one animal plays a minor role for detection of different resistance profiles [42].

MLST is well suited for epidemiological investigations, but may have a less discriminatory power for outbreak investigations than the pulsed-field gel electrophoresis (PFGE) [29]. Anyway, MLST may lead to more precise results for ESBL-producing *E*. *coli* than PFGE [43].

There are only a few studies investigating the prevalence of ESBL-producing bacteria in healthy livestock in Germany. In Bavaria, 39 of 45 cattle farms (86.%) tested positive for ESBL-producing *E*. *coli*, using fecal samples, dust samples and boot swabs [13]. Friese et al. examined animals from farms located in the northern and eastern part of Germany, finding a prevalence of 50% (n = 32) at pig farms, 60% (n = 10) at cattle farms and 100% (n = 6) at broiler farms [10]. The overall prevalence of their results is lower than in our study (60% vs 70%). In contrast to our results, all broiler farms were ESBL-positive, but their prevalence at pig farms was notably lower (50% vs 88%).

Usually, colonization rates in broiler flocks seem to increase during the fattening period [14, 44]. The dependence on age of the animals should be kept in mind when comparing different prevalence studies. In our study, the broiler farm with the youngest chickens (about one week old) turned out ESBL-negative, as did the two tested turkey farms, although the animals were at the end of the fattening period. The cloacal swabs identified no additional ESBL-positive poultry farms. On the contrary, at one poultry farm with an ESBL-positive boot swab, all sampled animals turned out to be negative. Therefore, the boot swabs seem more appropriate for identifying ESBL-positive barns. Nevertheless, the cloacal swabs gave additional information about the ESBL genes present in poultry barns.

Our study included four organic pig farms, all of which were positive for ESBL-producing *E*.*coli* in fecal samples. The number of tested organic farms was too small to be representative, and studies which examined the prevalence of ESBL- producing bacteria in organic farms are sparse. However, it demonstrates that ESBL-producing *E*. *coli* occur in organic pig farms.

The use of antibiotics may be an important factor for the distribution of multiresistant bacteria in livestock [29]. The usage of antimicrobials other than β-lactams may also lead to selection of ESBL-harboring bacteria [45]. Concerning ESBL-producing bacteria, other routes of acquiring resistance genes are also conceivable, e.g., even one-day-old broiler chickens can acquire ESBL-producing bacteria via vertical transmission from the parent or grandparent animals [44]. Therefore, trade is another important risk factor for the distribution of ESBL-producing *E*.*coli* [29].

The generally high occurrence of ESBL-producing *E*. *coli* in livestock demonstrated in this study allows the presumption of their broad dissemination in food producing animals. While a zoonotic transfer was not proven but would well fit to our results.

## Conclusion

Evidence for a direct transfer of ESBL-producing *E*. *coli* to persons working in close contact to livestock is sparse. This study implies that epidemiological links are likely to exist between livestock und farm workers. The high prevalence of ESBL-producing *E*. *coli* on pig, cattle and broiler farms in MP emphasizes the impact of livestock animals as a reservoir for such organisms. Further monitoring of the epidemiology of ESBL-producing bacteria in humans and livestock and the elucidation of possible transmission routes are needed.

## Supporting Information

S1 Table Partial sequences of the ESBL genes *bla*_CTX-M-1/-61_, *bla*_CTX-M-15/-28/-88_ and *bla*_TEM-104/-206._
(DOCX)

## References

[1] Chong Y, Ito Y, Kamimura T. Genetic evolution and clinical impact in extended-spectrum beta-lactamase-producing *Escherichia coli* and *Klebsiella pneumoniae* . Infect Genet Evol. 2011;11(7):1499–504. 10.1016/j.meegid.2011.06.001 21689785

[2] Falagas ME, Karageorgopoulos DE. Extended-spectrum beta-lactamase-producing organisms. J Hosp Infect. 2009;73(4):345–54. 10.1016/j.jhin.2009.02.021 19596491

[3] Pena C, Gudiol C, Tubau F, Saballs M, Pujol M, Dominguez MA, et al Risk-factors for acquisition of extended-spectrum beta-lactamase-producing *Escherichia coli* among hospitalised patients. Clin Microbiol Infect. 2006;12(3):279–84. 16451416 10.1111/j.1469-0691.2005.01358.x

[4] Canton R, Coque TM. The CTX-M beta-lactamase pandemic. Curr Opin Microbiol. 2006;9(5):466–75. 16942899 10.1016/j.mib.2006.08.011

[5] Ewers C, Bethe A, Semmler T, Guenther S, Wieler LH. Extended-spectrum beta-lactamase-producing and AmpC-producing *Escherichia coli* from livestock and companion animals, and their putative impact on public health: a global perspective. Clin Microbiol Infect. 2012;18(7):646–55. 10.1111/j.1469-0691.2012.03850.x 22519858

[6] Pitout JDD, Laupland KB. Extended-spectrum beta-lactamase-producing Enterobacteriaceae: an emerging public-health concern. Lancet Infectious Diseases. 2008;8(3):159–66. 10.1016/S1473-3099(08)70041-0 18291338

[7] Timofte D, Maciuca IE, Evans NJ, Williams H, Wattret A, Fick JC, et al Detection and molecular characterization of *Escherichia coli* CTX-M-15 and *Klebsiella pneumoniae* SHV-12 beta-lactamases from bovine mastitis isolates in the United Kingdom. Antimicrob Agents Chemother. 2014;58(2):789–94. 10.1128/AAC.00752-13 24247146 PMC3910873

[8] Teshager T, Dominguez L, Moreno MA, Saenz Y, Torres C, Cardenosa S. Isolation of an SHV-12 beta-lactamase-producing *Escherichia coli* strain from a dog with recurrent urinary tract infections. Antimicrob Agents Chemother. 2000;44(12):3483–4. 11185493 10.1128/aac.44.12.3483-3484.2000 PMC90231

[9] Brinas L, Moreno MA, Zarazaga M, Porrero C, Saenz Y, Garcia M, et al Detection of CMY-2, CTX-M-14, and SHV-12 beta-lactamases in *Escherichia coli* fecal-sample isolates from healthy chickens. Antimicrob Agents Chemother. 2003;47(6):2056–8. 12760899 10.1128/AAC.47.6.2056-2058.2003 PMC155838

[10] Friese A, Schulz J, Laube H, von Salviati C, Hartung J, Roesler U. Faecal occurrence and emissions of livestock-associated methicillin-resistant *Staphylococcus aureus* (laMRSA) and ESbl/AmpC-producing *E*. *coli* from animal farms in Germany. Berl Munch Tierarztl Wochenschr. 2013;126(3–4):175–80. 23540202

[11] Büchter B. Vorkommen und Charakterisierung von Extended-Spectrum-Beta-Laktamase (ESBL)-produzierenden *Escherichia coli* bei Lebensmittel liefernden Tieren, PhD-Thesis. Institut für Mikrobiologie und Tierseuchen des Fachbereichs Veterinärmedizin der Freien Universität Berlin; Bundesamt für Verbraucherschutz und Lebensmittelsicherheit, 2010.

[12] Wieler LH, Semmler T, Eichhorn I, Antao EM, Kinnemann B, Geue L, et al No evidence of the Shiga toxin-producing E. coli O104:H4 outbreak strain or enteroaggregative *E*. *coli* (EAEC) found in cattle faeces in northern Germany, the hotspot of the 2011 HUS outbreak area. Gut Pathog. 2011;3(1):17 10.1186/1757-4749-3-17 22051440 PMC3227623

[13] Schmid A, Hormansdorfer S, Messelhausser U, Kasbohrer A, Sauter-Louis C, Mansfeld R. Prevalence of extended-spectrum beta-lactamase-producing *Escherichia coli* on bavarian dairy and beef cattle farms. Appl Environ Microbiol. 2013;79(9):3027–32. 10.1128/AEM.00204-13 23455336 PMC3623142

[14] Laube H, Friese A, von Salviati C, Guerra B, Kasbohrer A, Kreienbrock L, et al Longitudinal monitoring of extended-spectrum-beta-lactamase/AmpC-producing *Escherichia coli* at German broiler chicken fattening farms. Appl Environ Microbiol. 2013;79(16):4815–20. 10.1128/AEM.00856-13 23747697 PMC3754693

[15] Moodley A, Guardabassi L. Transmission of IncN plasmids carrying blaCTX-M-1 between commensal *Escherichia coli* in pigs and farm workers. Antimicrob Agents Chemother. 2009;53(4):1709–11. 10.1128/AAC.01014-08 19188380 PMC2663060

[16] Hammerum AM, Larsen J, Andersen VD, Lester CH, Skovgaard Skytte TS, Hansen F, et al Characterization of extended-spectrum beta-lactamase (ESBL)-producing *Escherichia coli* obtained from Danish pigs, pig farmers and their families from farms with high or no consumption of third- or fourth-generation cephalosporins. J Antimicrob Chemother. 2014. 10.1093/jac/dku180 24908045

[17] Dierikx C, van der Goot J, Fabri T, van Essen-Zandbergen A, Smith H, Mevius D. Extended-spectrum-beta-lactamase- and AmpC-beta-lactamase-producing *Escherichia coli* in Dutch broilers and broiler farmers. J Antimicrob Chemother. 2013;68(1):60–7. 10.1093/jac/dks349 22949623

[18] Huijbers PM, Graat EA, Haenen AP, van Santen MG, van Essen-Zandbergen A, Mevius DJ, et al Extended-spectrum and AmpC beta-lactamase-producing *Escherichia coli* in broilers and people living and/or working on broiler farms: prevalence, risk factors and molecular characteristics. J Antimicrob Chemother. 2014;69(10):2669–75. 10.1093/jac/dku178 24879667

[19] Leverstein-van Hall MA, Dierikx CM, Cohen Stuart J, Voets GM, van den Munckhof MP, van Essen-Zandbergen A, et al Dutch patients, retail chicken meat and poultry share the same ESBL genes, plasmids and strains. Clin Microbiol Infect. 2011;17(6):873–80. 10.1111/j.1469-0691.2011.03497.x 21463397

[20] Leistner R, Meyer E, Gastmeier P, Pfeifer Y, Eller C, Dem P, et al Risk factors associated with the community-acquired colonization of extended-spectrum beta-lactamase (ESBL) positive *Escherichia Coli*. An exploratory case-control study. PLoS One. 2013;8(9):e74323 10.1371/journal.pone.0074323 24040229 PMC3770595

[21] Ewers C GM, Bethe A, Wieler LH, Guenthers S. Extended spectrum beta-lactamases producing Gram-negative bacteria in companion animals: action is clearly warrented! Berl Münch Tierärztliche Wochenschrift. 2011. 21462862

[22] Wang Y, He T, Han J, Wang J, Foley SL, Yang G, et al Prevalence of ESBLs and PMQR genes in fecal *Escherichia coli* isolated from the non-human primates in six zoos in China. Vet Microbiol. 2012;159(1–2):53–9. 10.1016/j.vetmic.2012.03.009 22487457

[23] Wallensten A, Hernandez J, Ardiles K, Gonzalez-Acuna D, Drobni M, Olsen B. Extended spectrum beta-lactamases detected in *Escherichia coli* from gulls in Stockholm, Sweden. Infect Ecol Epidemiol. 2011;1 10.3402/iee.v1i0.7030 22957123 PMC3426345

[24] Literak I, Dolejska M, Janoszowska D, Hrusakova J, Meissner W, Rzyska H, et al Antibiotic-resistant *Escherichia coli* bacteria, including strains with genes encoding the extended-spectrum beta-lactamase and QnrS, in waterbirds on the Baltic Sea Coast of Poland. Appl Environ Microbiol. 2010;76(24):8126–34. 10.1128/AEM.01446-10 20952638 PMC3008254

[25] Literak I, Dolejska M, Radimersky T, Klimes J, Friedman M, Aarestrup FM, et al Antimicrobial-resistant faecal *Escherichia coli* in wild mammals in central Europe: multiresistant *Escherichia coli* producing extended-spectrum beta-lactamases in wild boars. J Appl Microbiol. 2010;108(5):1702–11. 10.1111/j.1365-2672.2009.04572.x 19849769

[26] Guenther S, Grobbel M, Lubke-Becker A, Goedecke A, Friedrich ND, Wieler LH, et al Antimicrobial resistance profiles of *Escherichia coli* from common European wild bird species. Vet Microbiol. 2010;144(1–2):219–25. 10.1016/j.vetmic.2009.12.016 20074875

[27] Wirth T, Falush D, Lan R, Colles F, Mensa P, Wieler LH, et al Sex and virulence in *Escherichia coli*: an evolutionary perspective. Mol Microbiol. 2006;60(5):1136–51. 16689791 10.1111/j.1365-2958.2006.05172.x PMC1557465

[28] Fang H, Ataker F, Hedin G, Dornbusch K. Molecular epidemiology of extended-spectrum beta-lactamases among *Escherichia coli* isolates collected in a Swedish hospital and its associated health care facilities from 2001 to 2006. J Clin Microbiol. 2008;46(2):707–12. 18094139 10.1128/JCM.01943-07 PMC2238137

[29] Anonymous. Scientific opinion on the public health risks of bacterial strains producing extended-spectrum β-lactamases and/or AmpC β-lactamases in food and food-producing animals; EFSA Panel on Biological Hazards (BIOHAZ). EFSA Journal 2011;9(8):2322. 2011.

[30] Wu G, Day MJ, Mafura MT, Nunez-Garcia J, Fenner JJ, Sharma M, et al Comparative analysis of ESBL-positive isolates from animals and humans from the UK, the Netherlands and Germany. PLoS One. 2013;8(9):e75392 10.1371/journal.pone.0075392 24086522 PMC3784421

[31] Valentin L, Sharp H, Hille K, Seibt U, Fischer J, Pfeifer Y, et al Subgrouping of ESBL-producing *Escherichia coli* from animal and human sources: An approach to quantify the distribution of ESBL types between different reservoirs. Int J Med Microbiol. 2014. 10.1016/j.ijmm.2014.07.015 25213631

[32] Overdevest I, Willemsen I, Rijnsburger M, Eustace A, Xu L, Hawkey P, et al Extended-spectrum ß-lactamase genes of *Escherichia coli* in chicken meat and humans, the Netherlands. Emerg Infect Dis. 2011;17(7):1216–22. 10.3201/eid1707.110209 21762575 PMC3381403

[33] Brasme L, Nordmann P, Fidel F, Lartigue MF, Bajolet O, Poirel L, et al Incidence of class A extended-spectrum beta-lactamases in Champagne-Ardenne (France): a 1 year prospective study. J Antimicrob Chemother. 2007;60(5):956–64. 17804424 10.1093/jac/dkm319

[34] Mendonca N, Ferreira E, Louro D, Canica M. Molecular epidemiology and antimicrobial susceptibility of extended- and broad-spectrum beta-lactamase-producing *Klebsiella pneumoniae* isolated in Portugal. Int J Antimicrob Agents. 2009;34(1):29–37. 10.1016/j.ijantimicag.2008.11.014 19272757

[35] Hartmann A, Locatelli A, Amoureux L, Depret G, Jolivet C, Gueneau E, et al Occurrence of CTX-M Producing *Escherichia coli* in Soils, Cattle, and Farm Environment in France (Burgundy Region). Front Microbiol. 2012;3:83 10.3389/fmicb.2012.00083 22408639 PMC3297819

[36] Burt SA, Siemeling L, Kuijper EJ, Lipman LJ. Vermin on pig farms are vectors for *Clostridium difficile* PCR ribotypes 078 and 045. Vet Microbiol. 2012 10.1016/j.vetmic.2012.05.014 22682200

[37] Guenther S, Grobbel M, Beutlich J, Guerra B, Ulrich RG, Wieler LH, et al Detection of pandemic B2-O25-ST131 *Escherichia coli* harbouring the CTX-M-9 extended-spectrum beta-lactamase type in a feral urban brown rat (Rattus norvegicus). J Antimicrob Chemother. 2010;65(3):582–4. 10.1093/jac/dkp496 20071365

[38] Laube H, Friese A, von Salviati C, Guerra B, Rosler U. Transmission of ESBL/AmpC-producing *Escherichia coli* from broiler chicken farms to surrounding areas. Vet Microbiol. 2014;172(3–4):519–27. 10.1016/j.vetmic.2014.06.008 25035165

[39] Marathe NP, Regina VR, Walujkar SA, Charan SS, Moore ER, Larsson DG, et al A treatment plant receiving waste water from multiple bulk drug manufacturers is a reservoir for highly multi-drug resistant integron-bearing bacteria. PLoS One. 2013;8(10):e77310 10.1371/journal.pone.0077310 24204801 PMC3812170

[40] Valenza G, Nickel S, Pfeifer Y, Eller C, Krupa E, Lehner-Reindl V, et al Extended-spectrum-beta-lactamase-producing *Escherichia coli* as intestinal colonizers in the German community. Antimicrob Agents Chemother. 2014;58(2):1228–30. 10.1128/AAC.01993-13 24295972 PMC3910888

[41] Schauss T, Glaeser SP, Gutschow A, Dott W, Kampfer P. Improved detection of extended spectrum beta-lactamase (ESBL)-producing Escherichia coli in input and output samples of German biogas plants by a selective pre-enrichment procedure. PLoS One. 2015;10(3):e0119791 10.1371/journal.pone.0119791 25799434 PMC4370489

[42] Persoons D, Bollaerts K, Smet A, Herman L, Heyndrickx M, Martel A, et al The importance of sample size in the determination of a flock-level antimicrobial resistance profile for Escherichia coli in broilers. Microb Drug Resist. 2011;17(4):513–9. 10.1089/mdr.2011.0048 21875337 PMC3223426

[43] Nemoy LL, Kotetishvili M, Tigno J, Keefer-Norris A, Harris AD, Perencevich EN, et al Multilocus sequence typing versus pulsed-field gel electrophoresis for characterization of extended-spectrum beta-lactamase-producing Escherichia coli isolates. J Clin Microbiol. 2005;43(4):1776–81. 15814998 10.1128/JCM.43.4.1776-1781.2005 PMC1081380

[44] Dierikx CM, van der Goot JA, Smith HE, Kant A, Mevius DJ. Presence of ESBL/AmpC-producing *Escherichia coli* in the broiler production pyramid: a descriptive study. PLoS One. 2013;8(11):e79005 10.1371/journal.pone.0079005 24244401 PMC3820706

[45] Schwaber MJ, Navon-Venezia S, Schwartz D, Carmeli Y. High levels of antimicrobial coresistance among extended-spectrum-beta-lactamase-producing Enterobacteriaceae. Antimicrob Agents Chemother. 2005;49(5):2137–9. 15855548 10.1128/AAC.49.5.2137-2139.2005 PMC1087677

